# Composition of Sulla (*Hedysarum coronarium* L.) Honey Solvent Extractives Determined by GC/MS: Norisoprenoids and Other Volatile Organic Compounds

**DOI:** 10.3390/molecules15096375

**Published:** 2010-09-09

**Authors:** Igor Jerković, Carlo I.G. Tuberso, Mirko Gugić, Dragan Bubalo

**Affiliations:** 1 Faculty of Chemistry and Technology, University of Split, N. Tesle 10/V, 21000 Split, Croatia; 2 Dipartimento di Tossicologia, Università di Cagliari, via Ospedale 72, 09124 Cagliari, Italy; 3 Marko Marulić Polytechnic in Knin, P. Krešimira IV 30, 22300 Knin, Croatia; 4 Faculty of Agriculture, University of Zagreb, Svetošimunska 25, 10000 Zagreb, Croatia

**Keywords:** sulla (*Hedysarum coronarium* L.) honey, ultrasonic solvent extraction (USE), gas chromatography and mass spectrometry (GC and GC/MS), norisoprenoids

## Abstract

Samples of unifloral sulla (*Hedysarum coronarum* L.) honey from Sardinia (Italy) were analysed. To investigate the chemical composition of the honey volatiles two solvent systems were used for ultrasonic solvent extraction (USE): 1) a 1:2 (v/v) pentane and diethyl ether mixture and 2) dichloromethane. All the extracts were analysed by GC and GC/MS. These procedures have permitted the identification of 56 compounds that include norisoprenoids, benzene derivatives, aliphatic compounds and Maillard reaction products. Norisoprenoids were the major compounds in both extracts, dominated by vomifoliol (5.3-11.2%; 9.6-14.0%) followed by minor percentages of other norisoprenoids such as α-isophorone, 4-ketoisophorone, 3-oxo-α-ionol or 3-oxo-α-ionone. Other abundant single compounds in the extracts were 3-hydroxy-4-phenylbutan-2-one (0.8-5.4%; 0.6-5.7%) and methyl syringate (3.0-5.7%; 2.2-4.1%). The composition of the volatiles and semi-volatiles in the obtained extracts suggests that sulla honey is quite distinctive relative to the other honeys that have been chemically studied by GC/MS, but no specific markers of the honey botanical origin were found.

## 1. Introduction

Honeys produced from different floral sources may often have distinctly different aromas and tastes. During the last two decades, a lot of research has been done on the analysis of honey flavour volatile organic compounds. Some of these compounds derive from nectar, some are dependent on the physiology of the bee, and others arise during honey post-harvest processing as well as during storage. Methods of extracting honey volatiles may display a varying degree of selectivity and effectiveness, depending on the compounds involved [1,2,3,4]. Honey shake-flask liquid-liquid extraction with different solvents can be used for obtaining representative chemical composition of the volatiles without formation of thermal artefacts [4]. Ultrasonic solvent extraction (USE) has a major advantage over the previous method in significantly reducing extraction times [2,5]. There is mounting evidence that the organic solvent extractives of some unifloral honeys are chemically different from one another [6,7,8,9,10]. Different approaches in preparation of honey solvent extracts, prior to the GC and GC/MS analyses, have been reported: 1) a micro-scale simultaneous distillation-extraction (SDE) of the obtained solvent extract followed by the analysis of distillate [1]; 2) direct analysis of unmethylated extract [2,5]; 3) indirect analysis of methylated extract [7]. The chemical composition of honey solvent extracts obtained by 1) - 3) procedures revealed that the three main categories of natural volatiles are dominant in, or source specific for, honeys throughout the world: norisoprenoids, terpenes, and benzene derivatives [11]. Many researchers were focused on finding unique compounds to certify the botanical and geographical origin of different honeys and in many cases, such marker compounds seem to exist [11,12,13,14].

Sulla (*Hedysarum coronarium* L.) is a legume well adapted to semi-arid Mediterranean environments and represents an effective example of a multiple-uses species exploited for environmental protection, landscape enhancement and honey production [15]. Considerable amounts of sulla unifloral honey are produced in Southern and Central Italy. A few papers report general characteristics of sulla honey without detailed chemical analysis data. Honey from this species showed low invertase activity with an average value of 4.84 ± 2.26 SN [16]. In contrast, a high peroxide accumulation was obtained (45.25 ± 17.86 mg H_2_O_2_ g^-1^h^-1^). In addition, a chemometric approach to the discrimination of Italian honey samples from different floral origin [17] has been performed as a valuable classification tool to discriminate the botanical origin of six type of honey samples (chestnut, eucalyptus, heather, sulla, honeydew and wildflower).

Our preliminary research on sulla honey headspace by headspace solid-phase microextraction (HS-SPME) with fibers of different polarity as well as HPLC analyses of sulla honey ethyl acetate extracts did not reveal any useful data for characterization of this honey. Therefore, the scope of this work was to investigate the chemical composition of the organic extractives from unifloral sulla honey samples obtained by ultrasonic solvent extraction (USE) followed by GC and GC/MS analyses and to identify possible characteristic compounds for this honey type. Two extraction solvents were used: a pentane and diethyl ether (1:2 v/v) mixture and dichloromethane. To best of our knowledge, this is the first report on the honey volatiles composition of sulla.

## 2. Results and Discussion

**Figure 1:**
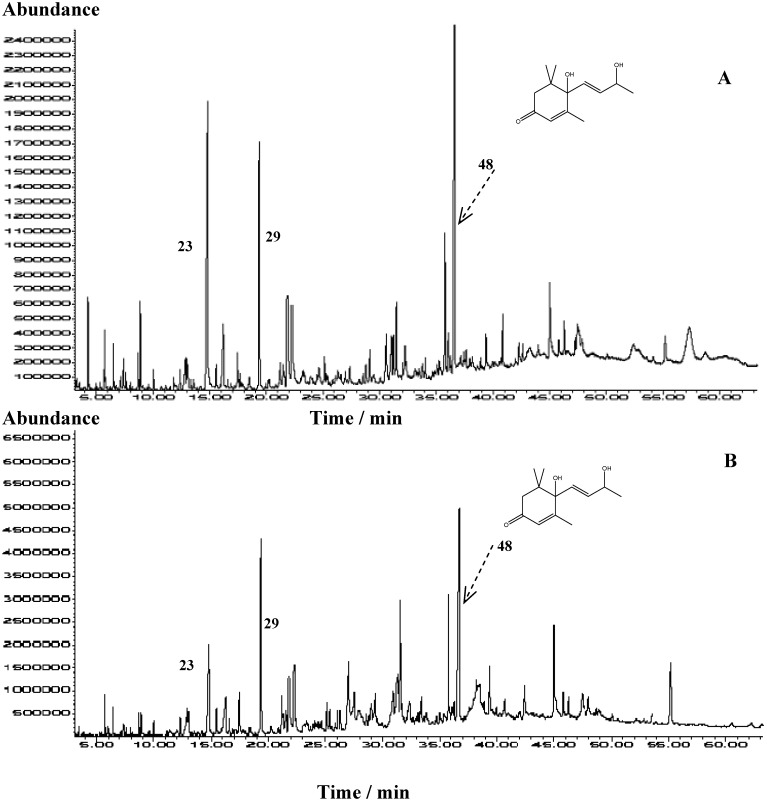
Representative TIC chromatograms of sulla honey extracts obtained by USE: **A -** dichloromethane extract; **B -** pentane and diethyl ether (1:2 v/v) extract. Numbers refer to Table 1.

Ultrasonic solvent extraction was applied for sulla honey samples since it reproducibly extracts natural honey volatiles and semi-volatiles (high-boiling volatiles) with good recoveries and without application of heat [5]. Comparison with other isolation methods this method is particularly appropriate for the isolation of water-soluble honey compounds [2]. To obtain a more complete composition two extraction solvents were used: A – a pentane and diethyl ether mixture and B - dichloromethane. Selected TIC chromatograms obtained from GC/MS analyses of USE extract using two solvents are presented in Figure 1. Great qualitative and quantitative similarity is observed among the extracts. Identified compounds are listed in Table 1 in accordance to their elution order on HP-5MS column. 

Norisoprenoids were the major group of identified organic compounds in both extracts (Table 1), dominated by vomifoliol (5.3-11.2%; 9.6-14.0%) followed by minor percentages of 3-oxo-α-ionol (0.2-1.5%; 1.0-1.2%), 3-oxo-α-ionone (0.0-2.9%; 1.1-1.9%), 3-oxo-7,8-dihydro-α-ionone (0.0-0.6%; 0.0%), 4-ketoisophorone (0.0-0.2%; 0.0-0.2%), α-isophorone (0.0-0.2%; 0.0-0.2%) and 6,7-dehydro-7,8-dihydro-3-oxo-α-ionol (0.0-0.6%; 0.0%). 

**Figure 2:**
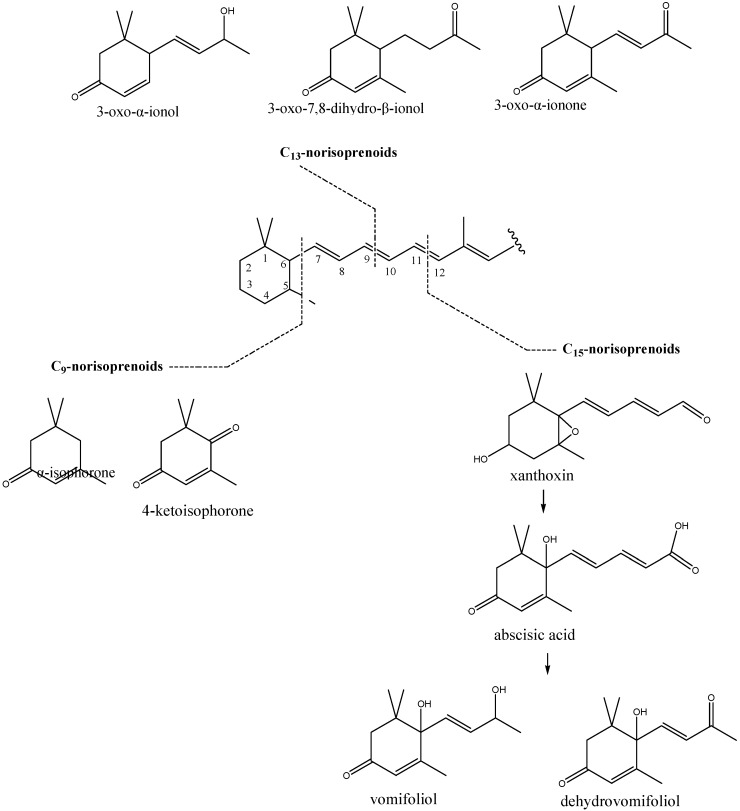
Different classes of degraded carotenoids and identified compounds in sulla honey from the classes of C_9_, C_13_ and C_15_ norisoprenoids.

Norisoprenoids are degraded-carotenoid-like structures (3,5,5-trimethyl-cyclohex-2-ene and its derivatives) and identified structures are presented in Figure 2.

In addition to the most widespread C_13_-norisoprenoids, volatile carotenoid metabolites with C_9_, C_10_ or C_11_ are also frequently detected in Nature [18]. The cleavage of the carotenoid chain is generally considered to proceed on different chain double bonds. Cleavage of C_11_-C_12_ double bond produces C_15_ norisoprenoids *via* abscisic acid, a well-known growth hormone, formed after cleavage of C_40_ carotenoids. *trans*,*cis*-Abscisic acid along with *trans*,*trans*-abscisic acid were found in New Zealand nectar honeys [9]. Zeaxanthin is the first committed abscisic acid precursor. A series of enzyme-catalyzed epoxidations and isomerisations, and final cleavage of the C_40_ carotenoid by a dioxygenation reaction yields precursor, xanthoxin, which is then further oxidized to abscisic acid [19]. Vomifoliol, the compound found in our extracts, probably arises through degradation of abscisic acid. Cleavage of C_9_-C_10_ double bond generates C_13_-norisoprenoids (such as 3-oxo-7,8-dihydro-β-ionol, 3-oxo-α-ionone or 3-oxo-α-ionol). Identified C_9_-norisoprenoids were α-isophorone and 4-ketoisophorone. Degraded carotenoid-like structures were aslo found in different honey extracts from New Zealand [9,10]. Volatile norisoprenoids were found as markers of botanical origin of Sardinian strawberry-tree honey [20]. Among them, α-isophorone, β-isophorone and 4-oxoisophorone were recognized as specific floral origin markers.

3-Hydroxy-4-phenylbutan-2-one (0.8-5.4%; 0.6-5.7%) is other major single compound in both sulla honey extracts belonging to the class of benzene derivatives raised from shikimic biogenetic pathway. However, 3-hydroxy-4-phenylbutan-2-one is probably not specific for any type of floral honey since it was previously found in the extracts from a range of different honeys [7,21,22]. Due to its previous isolation from flowers, 3-hydroxy-4-phenylbutan-2-one appears to originate from the plant nectar, and may contribute to the honey aroma, even though it appears not to be useful for the sourcing of the honeys. Methyl syringate (3.0-5.7%; 2.2-4.1%) is an abundant benzene derivative in the samples and was already detected in robinia, rape, chestnut, clover, linden blossom, dandelion, sunflower, thyme, manuka and fir honeys [23], but only in asphodel honey it reached the highest level [24]. Additionally, the results in Table 1 indicate great variability of benzene derivatives among the minor constituents of the volatiles extracted from the honey samples: ubiquitous benzaldehyde (0.0-0.1%; 0.1-0.2%), benzyl alcohol (0.1-0.3%; 0.0-0.3%), phenylacetaldehyde (0.0-0.2%; 0.3-0.5%) and 2-phenylethanol (0.2-0.4%; 0.2-0.5%), phenols [such as 2,4-dimethylphenol (0.3-0.4%; 0.3-0.5%)], aromatic acids [such as benzoic acid (0.4-4.6%; 0.4-3.9%), phenylacetic acid (1.2-5.8%; 1.0-4.8%), 4-methoxybenzoic acid (0.0-0.7%; 0.0-0.6%), cinnamic acid (0.0-0.9%; 0.0-0.6%), 4-methoxyphenylacetic acid (0.0-2.6%; 0.0-2.2%), α-hydroxyphenylpropanoic acid (0.0-25.7%; 0.0-1.4%) or 4-hydroxybenzoic acid (0.2-1.2%; 0.0-0.0%)] and others. 

Aliphatic compounds were also found in the extracts, being dominated by higher acids, alcohols and hydrocarbons such as hexadecanoic acid (0.9-2.5%; 0.5-1.1%), (*Z*)-octadec-9-en-1-ol (1.0-4.9%; 1.5-3.6%), hexadecan-1-ol (0.0-3.6%; 0.0-2.6%) or (*Z*)-octadec-9-enoic acid (0.0-2.4%; 0.0-1.2%). Detailed studies of these compounds in honey [10,25] indicate that they originate from beeswax and therefore are not useful as floral source descriptors.

Maillard reaction products (furan and pyran derivatives) were also found in minor percentages [such as 5-hydroxymethylfurfural (2.4–9.3%; 6.5-22.8%), 2,3-dihydro-3,5-dihydroxy-6-methyl-4*H*-pyran-4-one (0.3-0.6%; 0.2-0.9%), 2-methylfuran (0.2-1.2%; 0.2-2.3%) or dihydro-3-hydroxy-4,4-dimethyl-2(3*H*)-furanone (0.1-0.2%; 0.3-0.6%)], not as the honey markers but as indicators of absence of heat treatment and appropriate storage conditions.

## 3. Experimental 

### 3.1. Honey Samples

Fresh and unheated samples of sulla (*Hedysarum coronarium* L.) honey were collected from professional beekeepers in different areas of Sardinia (Italy). Sensory and melissopalynological analyses were used to attribute the floral origin and six samples were selected. Qualitative and quantitative melissopalynological analyses were carried out following the method of the International Commission of Bee Botany [26]. This involves the estimation of the absolute number of elements in the sediment, the identification of the most frequent elements, and the evaluation of the sulla pollen grains percentage.

### 3.2. Ultrasonic Solvent Extraction (USE)

Forty grams of each sample was diluted in distilled water (22 mL) in a 100-mL flask. Magnesium sulfate (1.5 g) was added and the flask was extensively vortexed. Two different extraction solvents were used: 1) mixture of pentane and diethyl ether (1:2 v/v) and 2) dichloromethane. The solvents were separately used for the extraction of each honey sample. Ultrasound-assisted solvent extraction (USE) was performed in an ultrasound cleaning bath (Elmasonic Typ S 30 H, Germany) by indirect sonication (sweep mode), at the frequency of 37 kHz at 25 ± 3 °C. Sonication was maintained for 30 min. After sonication, the organic layer was separated by centrifugation and filtered over anhydrous MgSO4. The aqueous layer was returned to the flask and another batch of the same extraction solvent (20 mL) was added and extracted by ultrasound for 30 min. The organic layer was separated in the same way as the previous one and filtered over anhydrous MgSO4, and the aqueous layer was sonicated a third time for 30 min with another batch (20 mL) of the extraction solvent. Joined organic extracts were concentrated to 0.2 mL by distillation with Vigreaux column, and 1 μL was used for GC and GC/MS analyses. For each sample, three replicates were obtained.

### 3.3. Gas Chromatography and Mass Spectrometry (GC, GC/MS)

Gas chromatography analyses were performed using an Agilent Technologies (Palo Alto, CA, USA) gas chromatograph model 7890A equipped with flame ionization detector, quadropol mass spectrometer model 5975C equipped with the capillary column HP-5MS ((5%-phenyl)-methylpolysiloxane Agilent J & W GC column, 30 m, 0.25 mm i.d., coating thickness 0.25 μm). Chromatographic conditions were as follows: helium was carrier gas at 1 mL·min^−1^, injector temperature was 250 °C, and FID detector temperature was 300 °C. The temperature used included the following settings: 70 °C isothermal for 2 min, and then increased to 200 °C at a rate of 3 °C·min^−1^ and held isothermal for 18 min. The injected volume was 1 μL and the split ratio was 1:50. Mass spectra were recorded in the electron ionization mode at 70 eV with ion source temperature 230 °C and scanning the 30-300 *m/z* range. The analyses were carried out in duplicate for each sample batch.

The individual peaks were identified by comparison of their retention indices (relative to C9-C25 *n-*alkanes for HP-5MS) to those of available authentic samples and literature [27], as well as by comparing their fragmentation pattern with the Wiley 275 MS library (Wiley, New York, NY, USA) and NIST02 (Gaithersburg, MD, USA) mass spectral database. The percentage composition of the samples was computed from the GC peak areas using the normalization method (without correction factors). The component percentages were calculated as mean values from duplicate GC and GC-MS analyses.

## 4. Conclusions

There is moderate diversity among the natural volatiles that are extractable from Sardinian sulla honey samples, including a variety of distinctive norisoprenoids, benzene derivatives, aliphatic compounds and Maillard reaction products, but only a few terpenes were found. Both extraction solvents revealed very similar chemical composition and therefore can both be used for the isolation of sulla honey volatiles. Sulla honey is characterized by high percentages of norisoprenoids, being dominated by vomifoliol. Other major compounds were 3-hydroxy-4-phenylbutan-2-one and methyl syringate. The composition of the volatiles and semi-volatiles in the obtained extracts suggests that sulla honey is quite distinctive relative to the other honeys that have been chemically studied by GC/MS, but no specific markers of the honey botanical origin were found. However the obtained results present good starting point for chemical characterization of the sulla honey, since no useful data were obtained by HPLC and HS-SPME in our preliminary study. 

## Figures and Tables

**Table 1:** Sulla (*Hedysarum coronarium* L.) honey volatile organic composition obtained by USE with two solvents followed by GC and GC/MS analysis.

No.	Compound	RI	Area percentage (%)								
			Solvent A					Solvent B			
			Min.	Max.	Av.	SD.		Min.	Max.	Av.	SD.
1.	2-Furanmethanol	< 900	0.0	0.1	0.05	0.06		-	-	-	-
2.	2-Methylbutanoic acid	< 900	0.0	0.1	0.05	0.06		-	-	-	-
3.	Hexan-1-ol	< 900	0.0	0.1	0.08	0.05		-	-	-	-
4.	1,4-Dimethylbenzene^**^	< 900	0.0	0.2	0.10	0.08		-	-	-	-
5.	Benzaldehyde	965	0.0	0.1	0.05	0.06		0.1	0.2	0.13	0.05
6.	2,4-Dimethyl-3,6-dihydro-2*H*-pyran	973	0.0	0.1	0.05	0.06		-	-	-	-
7.	2-Methylfuran^*^	988	-	-	-	-		0.2	2.3	0.98	0.93
8.	2-Hydroxy-3-methylcyclopent-2-en-1-one	1036	-	-	-	-		0.0	0.1	0.05	0.06
9.	Benzyl alcohol	1037	0.1	0.3	0.18	0.09		0.0	0.3	0.15	0.13
10.	Dihydro-3-hydroxy-4,4-dimethyl-2(3*H*)-furanone (Pantolactone)	1044	0.1	0.2	0.15	0.06		0.3	0.6	0.43	0.15
11.	Phenylacetaladehyde	1048	0.0	0.2	0.13	0.10		0.3	0.5	0.28	0.17
12.	4,5-Dimethyl-2-formylfuran	1078	0.4	0.8	0.40	0.29		0.3	0.7	0.26	0.26
13.	Methyl 2-furoate	1084	0.0	0.4	0.18	0.21		0.0	1.5	0.55	0.71
14.	1-(2-Furanyl)-2-hydroxyethanone	1088	0.2	0.7	0.45	0.21		0.8	2.2	1.23	0.66
15.	2-Phenylethanol	1116	0.2	0.4	0.30	0.12		0.2	0.5	0.35	0.13
16.	3,5,5-Trimethyl-cyclohex-3-en-1-one (α-Isophorone)	1124	0.0	0.2	0.08	0.10		0.0	0.2	0.10	0.08
17.	2,3-Dihydro-3,5-dihydroxy-6-methyl-4*H*-pyran-4-one	1145	0.3	0.6	0.43	0.15		0.2	0.9	0.48	0.31
18.	3,5,5-Trimethyl-cyclohex-2-ene-1,4-dione (4-Ketoisophorone)	1147	0.0	0.2	0.10	0.08		0.0	0.2	0.10	0.08
19..	Benzoic acid	1162	0.4	4.6	1.73	1.95		0.4	3.9	1.48	1.67
20.	2,4-Dimethylphenol^**^	1181	0.3	0.4	0.33	0.10		0.3	0.5	0.33	0.13
21.	(*E*)-3,7-Dimethyl-octa-1,5-diene-3,7-diol	1191	0.0	0.4	0.20	0.23		0.3	0.6	0.45	0.13
22.	Decanal	1207	0.0	0.2	0.08	0.10		0.0	0.1	0.05	0.06
23.	5-Hydroxymethylfurfural	1230	2.4	9.3	4.78	3.08		6.5	22.8	13.03	6.91
24.	1,3-Bis(1,1-dimethylethyl)benzene	1261	0.0	0.7	0.23	0.33		-	-	-	-
25.	Phenylacetic acid	1269	1.1	5.8	2.90	2.02		1.0	4.8	2.53	1.62
26.	Nonanoic acid	1273	0.0	0.2	0.08	0.10		-	-	-	-
27.	1-Methoxy-4-propylbenzene	1305	0.0	0.5	0.18	0.24		0.0	0.8	0.40	0.34
28.	2,4,6-Trimethylphenol^**^	1332	0.0	0.6	0.28	0.32		0.0	0.4	0.13	0.19
29.	3-Hydroxy-4-phenylbutan-2-one	1354	0.8	5.4	3.73	2.02		0.6	5.7	3.43	2.15
30.	1-(4-Methoxyphenyl)-ethanone (4-Methoxyacetophenone)	1360	0.0	0.2	0.08	0.10		-	-	-	-
31.	(*E*)-8-Hydroxylinalool	1367	0.0	1.1	0.33	0.53		0.2	0.3	0.23	0.05
32.	Tetradecane	1400	0.0	0.8	0.28	0.38		-	-	-	-
33.	4-Hydroxyphenyl ethanol	1445	0.0	1.2	0.80	0.57		0.0	0.4	0.18	0.21
34.	4-Methoxybenzoic acid	1451	0.0	0.7	0.23	0.33		0.0	0.6	0.33	0.25
35.	3-Phenylprop-2-enoic acid^**^ (Cinnamic acid)	1454	0.0	0.9	0.40	0.38		0.0	0.6	0.33	0.25
36.	4-Methoxyphenylacetic acid	1496	0.0	2.6	0.85	1.23		0.0	2.2	0.73	1.04
37.	Pentadecane	1500	0.0	0.5	0.20	0.25		-	-	-	-
38.	4-Methyl-2,6-bis(1,1-dimethylethyl)-phenol	1514	1.1	3.0	1.83	0.82		0.0	0.1	0.05	0.06
39.	α-Hydroxyphenylpropanoic acid	1545	0.0	15.7	5.25	7.40		0.0	1.4	0.48	0.66
40.	2-Hydroxydecanoic acid	1557	0.0	2.0	0.78	0.86		0.0	0.6	0.33	0.25
41.	4-Hydroxybenzoic acid	1558	0.2	1.2	0.55	0.55		-	-	-	-
42.	*cis*-*p*-Menth-8-ene^*^	1632	0.0	0.9	0.38	0.45		0.0	1.7	0.68	0.73
43.	3-Oxo-α-ionol	1660	0.2	1.5	0.83	0.56		1.0	1.2	1.08	0.10
44.	3-Oxo-α-ionone	1665	0.0	2.9	1.90	1.32		1.1	1.9	1.60	0.38
45.	3-Oxo-7,8-dihydro-α-ionone	1682	0.0	0.6	0.38	0.26		-	-	-	-
46.	6,7-Dehydro-7,8-dihydro-3-oxo-α-ionol	1720	0.0	0.6	0.25	0.30		-	-	-	-
47.	Methyl syringate	1744	3.0	5.7	4.38	1.15		2.2	4.1	3.28	0.79
48.	6-Hydroxy-3-oxo-α-ionol (Vomifoliol)	1802	5.3	11.2	8.88	2.62		9.6	14.0	11.63	1.84
49.	Hexadecan-1-ol	1882	0.0	3.6	1.50	1.51		0.0	2.6	1.18	1.07
50.	Nonadecane	1900	0.0	0.7	0.33	0.38		-	-	-	-
51.	Hexadecanoic acid	1963	0.9	2.5	1.43	0.73		0.5	1.1	0.75	0.25
52.	(*Z*)-Octadec-9-en-1-ol	2060	1.0	4.9	2.55	1.95		1.5	3.6	2.70	0.98
53.	Octadecan-1-ol	2084	0.0	0.8	0.50	0.36		-	-	-	-
54.	Heneicosane	2100	0.0	0.6	0.20	0.28		-	-	-	-
55.	(*Z*)-Octadec-9-enoic acid	2147	0.0	2.4	1.30	1.10		0.0	1.2	0.58	0.53
56.	Tetracosane	2400	0.0	3.1	1.60	1.68		0.0	3.3	1.85	1.37

RI = retention indices on HP-5MS column; A = USE with pentane and diethyl ether (1:2 v/v) mixture; B = USE with dichloromethane; - = not detected; ^*^ - tentatively identified; ^**^ - correct isomer not identified.

## Supplementary Materials

Supplementary File 1
