# Correction: Jerković, I., *et al*. Composition of Sulla (*Hedysarum coronarium* L.) Honey Solvent Extractives Determined by GC/MS: Norisoprenoids and Other Volatile Organic Compounds. *Molecules* 2010, *15*, 6375-6385

**DOI:** 10.3390/molecules181113434

**Published:** 2013-10-30

**Authors:** Igor Jerković, Carlo I. G. Tuberoso, Mirko Gugić, Dragan Bubalo

**Affiliations:** 1 Faculty of Chemistry and Technology, University of Split, N. Tesle 10/V, 21000 Split, Croatia; 2 Dipartimento di Tossicologia, Università di Cagliari, via Ospedale 72, 09124 Cagliari, Italy; 3 Marko Marulić Polytechnic in Knin, P. Krešimira IV 30, 22300 Knin, Croatia; 4 Faculty of Agriculture, University of Zagreb, Svetošimunska 25, 10000 Zagreb, Croatia

The authors wish to make the following correction to paper [1], doi:10.3390/molecules15096375, website: http://www.mdpi.com/1420-3049/15/9/6375.

The correct name of the second author is: Carlo I. G. Tuberoso.

## References

[1] Jerković I., Tuberso C.I.G., Gugić M., Bubalo D. (2010). Composition of sulla (*Hedysarum coronarium* L.) honey solvent extractives determined by GC/MS: Norisoprenoids and other volatile organic compounds. Molecules.

