# Supplementary material for: Composition of Sulla (Hedysarum coronarium L.) Honey Solvent Extractives Determined by GC/MS: Norisoprenoids and Other Volatile Organic Compounds
Source: Molecules. 2010 Sep 9;15(9):6375–85. doi: 10.3390/molecules15096375 (PMC6257696; doi:10.3390/molecules15096375)
Supplement: Supplementary File 1 [file molecules-15-06375-s001.pdf]

Molecules **2013**, *18*, 13434; doi:10.3390/molecules181113434

OPEN ACCESS

molecules

ISSN 1420-3049

www.mdpi.com/journal/molecules

Correction

Correction: Jerković, I., *et al*. Composition of Sulla (*Hedysarum coronarium* L.) Honey Solvent Extractives Determined by GC/MS: Norisoprenoids and Other Volatile Organic Compounds. *Molecules* 2010, *15*, 6375-6385

Igor Jerković [1,]*, Carlo I. G. Tuberoso [2], Mirko Gugić [3] and Dragan Bubalo [4]

[1] Faculty of Chemistry and Technology, University of Split, N. Tesle 10/V, 21000 Split, Croatia
[2] Dipartimento di Tossicologia, Università di Cagliari, via Ospedale 72, 09124 Cagliari, Italy
[3] Marko Marulić Polytechnic in Knin, P. Krešimira IV 30, 22300 Knin, Croatia
[4] Faculty of Agriculture, University of Zagreb, Svetošimunska 25, 10000 Zagreb, Croatia

* Author to whom correspondence should be addressed; E-Mail: igor@ktf-split.hr.

Received: 3 September 2013; in revised form: 21 October 2013 / Accepted: 30 October 2013 / Published: 30 October 2013

The authors wish to make the following correction to paper [1], doi:10.3390/molecules15096375, website: http://www.mdpi.com/1420-3049/15/9/6375.

The correct name of the second author is: Carlo I. G. Tuberoso.

Reference

1. Jerković, I.; Tuberso, C.I.G.; Gugić, M.; Bubalo, D. Composition of sulla (*Hedysarum coronarium* L.) honey solvent extractives determined by GC/MS: Norisoprenoids and other volatile organic compounds. *Molecules* **2010**, *15*, 6375–6385.

© 2013 by the authors; licensee MDPI, Basel, Switzerland. This article is an open access article distributed under the terms and conditions of the Creative Commons Attribution license (http://creativecommons.org/licenses/by/3.0/).
